# Evaluation of the impact of vindoline, an active components of *Catharanthus roseus*, on rat hepatic cytochrome P450 enzymes by using a cocktail of probe drugs

**DOI:** 10.1371/journal.pone.0289656

**Published:** 2023-08-03

**Authors:** Yuqian Zhang, Haiying Niu, Jian Liu, Weiwei Xie, Yiran Jin, Zhiqing Zhang

**Affiliations:** 1 The Second Hospital of Hebei Medical University, Shijiazhuang, Hebei, P. R. China; 2 The First Hospital of Hebei Medical University, Shijiazhuang, Hebei, P. R. China; Foshan University, CHINA

## Abstract

The objection of this study was to investigate the effects of vindoline(VDL) on the cytochrome P450 (CYP 450) isoforms (CYP1A2, 2B, 2C11, 2D1 and 3A) in rats. Firstly, the rats were randomly divided into VDL pretreatment group and blank group, each group had six rats. VDL pretreatment group was administrated VDL (20 mg·kg^-1^) by oral gavage for fifteen days consecutively, and the equivalent CMC-Na solution without VDL was given to the blank group by gavage. Secondly, a cocktail of caffeine, bupropion, diclofenac, dextromethorphan and midazolam was then administered on the sixteenth day. Finally, blood samples were collected at the specified time point, and the plasma concentration of the probe drug was determined by UHPLC-QTOF-MS/MS. The effects of VDL on the activity of these CYP enzymes in rats were evaluated by pharmacokinetic parameters. VDL pretreatment group compared with the blank group, accelerated the metabolism of diclofenac, and weakened the metabolism of caffeine. These results suggested that VDL could induce the activity of CYP2C11, and inhibits the activity of CYP1A2, but had no significant effects on CYP2B, CYP2D1 and CYP3A. The results in this study can provide beneficial information for the later clinical application of VDL.

## 1. Introduction

*Catharanthus roseus (L*.*) G*. *Don* (*C*. *roseus*), an aboriginal perennial plant species of Madagascar, is often known as the Madagascar periwinkle [1, 2]. *C*. *roseus* is a tropical perennial subshrub known to produce about 130 alkaloids, such as vinblastine, vincristine, vinleurosine, vindoline, and catharanthine [3, 4]. In some countries, leaf and whole plant decoctions are used as home remedies for diabetes [5, 6]. Previous literature shows that the *C*. *roseus* leaf juice has significant antidiabetes activity, which may help prevent diabetes complications and play a good auxiliary role in the current antidiabetes drugs [5, 7]. Vindoline (VDL) is a semi-synthetic intermediate vinca alkaloid, which can be extracted from *C roseus*, it is reported to have anti diabetic properties in diabetes induced animal models [8, 9]. Recently, it was reported that VDL has the inhibitory effect on protein tyrosine phosphatase 1B (PTP-1B), so it can be used as an "insulin sensitizer" in the treatment of type 2 diabetes mellitus (T2DM) [10–12].

Diabetes is a lifelong metabolic diseases characterized by chronic hyperglycemia due to multiple etiologies [13]. According to the World Health Organization (WHO), diabetes has up to 100 complications, and which is currently one of the most known complications [14]. More than half of the deaths from diabetes were due to cardio cerebrovascular causes, and 10% were due to renal lesions. Clinical data shows that 30% - 40% of patients with diabetes will have at least one complication about 10 years after the onset of diabetes [15]. So diabetic patients usually take hypoglycemic drugs for a long time and even suffer from lifelong drug treatment, and it is often in combination with other drugs [16]. Taking multiple drugs at the same time can easily create drug-drug interactions (DDIs). As a potential hypoglycemic agent, VDL may be used in combination with other drugs. Therefore, it is necessary to clarify the effects of VDL on CYP enzymes activity [17].

The cytochrome P450 (CYP450) enzyme system has the greatest relationship with drug metabolism [18]. It mainly exists in human liver, and also has a small amount of expression in kidney, small intestine, lung and brain [19]. It participates in the biological transformation of many endogenous and exogenous substances (including most clinical drugs) of organisms [20]. DDIs should be considered in clinical use, mainly because there are many drugs that can enhance or weaken the activity of CYP (inducer), change the rate of drug metabolism, and affect the effect of drugs [21].

Recently a quadrupole time-of-flight mass (TripleTOF-MS) spectrometer has been routinely used as an effective and reliable analytical instrument [22]. The TripleTOF 5600+ system has a new ultra-high sensitivity designs, it can achieve high resolution and open a very narrow detection window without decreasing the sensitivity. At this time, it eliminates the interference of various impurities and has high selectivity. Therefore, the quantitative sensitivity can be comparable to the MRM sensitivity of API 4000 or API 5000; at the same time, the scanning speed as high as 100 spectra/s ensures that sufficient data points can be collected even for the peak width of UHPLC in a few seconds, so as to obtain excellent reproducibility and reliability.

Theoretically, human CYP1A2 is homolog to rat CYP1A2, human CYP2B is homolog to rat CYP2B, human CYP2C9 is homolog to rat CYP2C11, human CYP2D6 is homolog to rat CYP2D1 and human CYP3A4 is homolog to rat CYP3A [20, 23]. So rats were chosen as experimental animal in this research. To investigate the effects of VDL on rat hepatic CYP enzymes, this study adopted a probe cocktail approach using caffeine (CYP1A2), bupropion (CYP2B), dextromethorphan (CYP2C11), diclofenac acid (CYP2D1) and midazolam (CYP3A). Meanwhile, an ultra-high-performance liquid chromatography–tandem mass spectrometry (UHPLC–MS) method was used to detect these five probe drugs, which can provide a highly selective and sensitive assay with low limits of quantification.

## 2. Experimental

### 2.1 Chemicals and materials

The reference standards (purity>98%) of VDL (CAS:2182-14-1) were purchased from Shanghai Shifeng Biological Technology Co. Ltd. Caffeine, diclofenac sodium (purity≥99%) were purchased from China National Food and Drug Administration; Bupropion hydrochloride (purity≥98%) was purchased from Tokyo Institute of Physics and Chemistry; Dextromethorphan hydrobromide (purity≥98%) and phenacetin (purity≥99%) were purchased from Dalian Meilun Biotechnology Co., Ltd.; Midazolam (purity>98.0%) was purchased from J&KScientific Ltd. (Beijing, China). HPLC-grade acetonitrile and LC-MS grade formic acid were obtained from Fisher Chemicals (USA). Heparin Sodium Injection (for heparinizing centrifuge tubes) was purchased from Tianjin Biochem Pharmaceutical co., Ltd. (Tianjin, China). The other chemicals were all of analytical-reagent grade.

### 2.2 Instrumentations

Samples were analyzed by UHPLC-QTOF-MS/MS, which consisted of a Prominence^TM^ UHPLC System (Shimadzu, Japan) and a Triple TOF^TM^ 5600+ system (AB SCIEX, USA) equipped with Duo-Spray^TM^ ion sources in the electrospray ionization (ESI) technology.

### 2.3 Chromatographic conditions

The samples were separated by a Phenomenexkinetex C18 (3.0×50 mm, 2.6 μm), the column temperature was 40°C. The mobile phase consisted of 0.1% formic acid (A) and acetonitrile (B) at a flow rate of 400 μL·min^-1^, the gradient elution program was established as 0–0.5 min, 20% B; 0.5–4 min, 20–95% B; 4–5 min, 95% B; 5–5.1 min, 95–20% B. The injection volume was 2 μL.

### 2.4 Mass spectrometric conditions

The mass detection was accomplished in multiple reaction monitoring (MRM^HR^) mode. Analyst^®^ TF 1.7 software (AB SCIEX, USA) and MultiQuant 3.0.1 (AB SCIEX, USA) were used for instrument control, data acquisition as well as data processing. The ESI source was set to positive ion mode with ionization conditions as follows: ion spray voltage floating, (+) 5.5 kV; ion source heater, 550°C; curtain gas, 25 psi; ion source gas 1, 55 psi; and ion source gas 2, 55 psi. The detected ions and collision energy (CE) of the five cocktail probe drugs are shown in Table 1. The collision energy expansion (CES) is 0 eV. A narrow extraction window (±0.02 Da) was used in MultiQuant 3.0.1 to leverage the high resolution data.

**Table 1:** The CE and detected ions of five cocktail probe drugs and IS.

Isoform	Probe	Formula	CE	Detected ion
CYP1A2	Caffeine	C₈H_10_N₄O₂	20	195.1905±0.02 Da
CYP2B	Bupropion	C_13_H_18_ClNO	15	184.0528±0.02 Da
CYP2C11	Diclofenac	C_14_H_11_Cl_2_NO_2_	25	250.0178±0.02 Da
CYP2D1	Dextromethorphan	C_18_H_25_NO	38	147.0818±0.02 Da
CYP3A	Midazolam	C_18_H_13_ClFN_3_	40	291.1150±0.02 Da
IS	Phenacetin	C_10_H_13_NO_2_	30	110.0608±0.02 Da

### 2.5 Standard and sample preparation

#### 2.5.1 Preparation of calibration curve samples and quality control (QC) samples

Five probe drug stock solutions with a concentration of 1mg/mL were prepared in methanol. A series of working solutions were obtained by diluting the stock solution with methanol. Additionally, the phenacetin (IS) working solution was obtained by diluting the stock standard solution with methanol to yield the final concentration of 0.2 μg/mL. All the solutions were stored at 4°C until analysis.

Add the working solution into 100 μL blank plasma to prepare the calibration standards and quality control (QC) samples. The final plasma concentrations of calibration samples were adjusted to 3.007, 15.015, 30.07, 60.24, 300.7, 602.4, 1501.5, 3007 ng/mL for caffeine; 0.3075, 1.537, 3.075, 6.150, 30.75, 61.50, 153.7, 307.5 ng/mL for bupropion; 0.2032, 1.016, 2.032, 4.064, 20.32, 40.64, 101.6, 203.2 ng/mL for dextromethorphan; 2.907, 14.535, 29.07, 58.14, 290.7, 581.4, 1453.5, 2907 ng/mL for diclofenac; 0.5000, 2.500, 5.000, 10.00, 50.00, 100.0, 250.0, 500.0 ng/mL for midazolam. Quality control (QC) samples, including low, medium, high concentration and lower limit of quantification (LLOQ) levels, with final concentrations of 7.962, 300.7, 2255, 3.007 ng/mL for caffeine; 0.7688, 30.75, 230.6, 0.3075 ng/mL for bupropion; 7.267, 290.7, 2180, 2.907 ng/mL for diclofenac; 0.5080, 20.32, 152.4, 0.2032 ng/mL for dextromethorphan; 1.250, 50.00, 375.0, 0.500 ng/mL for midazolam.

#### 2.5.2 Sample preparation

10 μL of methanol and 10 μL of IS working solution were added into 100 μL of rat plasma sample. Then adding 300 acetonitrile to precipitate protein, the mixture was vortexed for 5min, and centrifuged at 15000 rpm for 10min, the supernatant was transferred to another tube, evaporated to dryness under a stream of nitrogen. The residue was reconstituted with 150 μL of 80% acetonitrile and centrifuged at 15000 rpm for 10 min. Before injection, 2 μL of supernatant was used for UHPLC-QTOF-MS/MS analysis.

### 2.6 Application of the analytical method in CYP450 activity study

Male Sprague–Dawley rats (Certificate No.SCXK 2013-1-003; weighting 250±20g; 7–8 weeks of age) were supplied by the Experimental Animal Center of Hebei Laboratory Animal Center (Shijiazhuang, China). Before the experiment, rats were fed in a breeding room with a temperature of 22–25° C, a relative humidity of 55–60% and a 12h light/dark cycle for a week. During this period, rats ate and drank normally, and fasted overnight before administration. All experiments on rats were conducted in accordance with the guidelines of the Committee on the Care and Use of Laboratory Animals in our laboratory.

The rats were randomly divided into two groups: the VDL pretreatment group and the blank group, six rats in each group. The blank group took 5% CMC-Na solution orally for 15 days; the VDL treated groups were administered quantitatively VDL (20mg·kg^-1^) [7] for 15 consecutive days; On the sixteenth day, the VDL treated group and the blank group were administrated mixed cocktail probe drugs, which included caffeine (5mg·kg^-1^; for CYP1A2 activity), bupropion (5 mg·kg^-1^; for CYP2B activity); diclofenac (5mg·kg^-1^; for CYP2C11 activity), dextromethorphan (5mg·kg^-1^; for CYP2D1 activity) and midazolam(5 mg·kg^-1^; for CYP3A activity). Blood samples were collected from the epicanthic veins at 0 (pre-dose), 0.08 (5 min), 0.25 (15 min), 0.5 (30 min), 0.75 (45 min), 1, 1.5, 2, 3, 4, 6, 8, 12, 24 h, and transfered it to the heparinized centrifuge tube. Then the tubes with blood samples centrifuged at 4500 *g* for 5 min, and transfer 0.1mL of plasma layer into a clean centrifuge tube and store it at -80°C. At the end of this study, the animals were anesthetized under 3% isoflurane and euthanized by exsanguination via cardiac puncture [24].

### 2.7 Statistical analysis

The pharmacokinetic parameters of the five probe drugs were derived with a non-linearre gression iterative program, and calculated with the DAS 3.0 pharmacokinetic program (Chinese Pharmacological Society, Beijing, China). The statistical analysis used SPSS 21.0 (IBM SPSS, USA), and compared the pharmacokinetic characteristics of five probe drugs with One-way analysis of variance (ANOVA). When *P*<0.05, the difference was considered statistically significant.

## 3. Results

An efficient and reliable UHPLC-MS method for simultaneous determination of caffeine, bupropion, diclofenac, dextromethorphan, and midazolam is established, thus can be applied to activity evaluation of CYP1A2, 2B, 2C11, 2D1 and 3A with a cocktail approach.

### 3.1 Method validation

**Fig 1:**
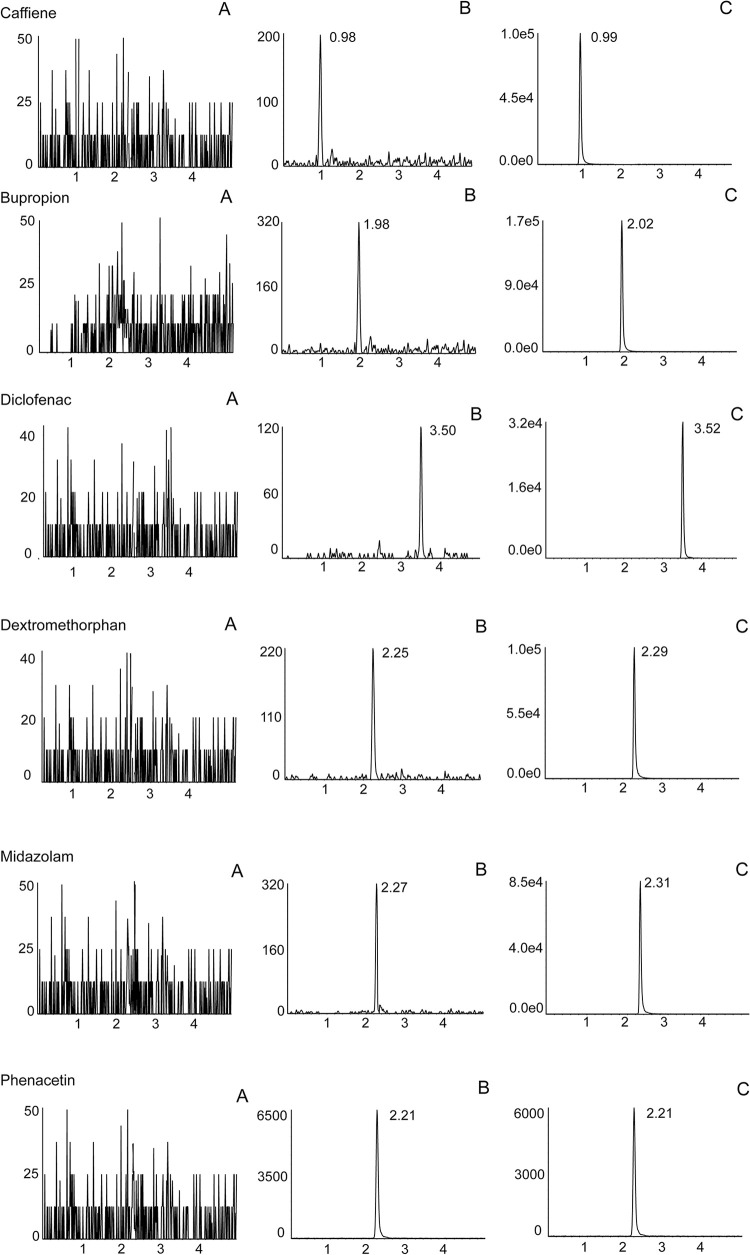
Chromatograms of cocktail probe drugs and IS in positive ion mode. Note: (A) blank plasma; (B) blank plasma spiked with cocktail probe drugs at LLOQ and IS; (C) sample plasma 1 h after administration of cocktail solution.

The chromatograms of the blank plasma samples, blank plasma samples spiked with analytes at LLOQ and plasma samples from rats 1h after oral administration of cocktail solution are shown in Fig 1. The calibration curves were obtained by using a weighting factor of 1/x^2^. The calibration curves, linear ranges, correlation coefficients and LLOQs of the five cocktail probe drugs are shown in Table 2. The real values of each calibration concentration were between 89% and 112% of the theoretical values. None of the RE and RSD values of the intra-day and inter-day accuracy and precision exceeded 15%.

**Table 2:** The regression equations, linear ranges, r and LLOQs of the five cocktail probe drugs.

Compounds	Regression equation	*r*	Linear range (ng/mL)	LLOQ (ng/mL)
Caffeine	*Y* = 0.0177*X*+0.0092	0.9973	3.007–3007	3.007
Bupropion	*Y* = 0.0431*X*+0.0196	0.9980	0.3075–307.5	0.3075
Diclofenac	*Y* = 0.0161*X*+0.0084	0.9931	2.907–2907	2.907
Dextromethorphan	*Y* = 0.0515*X*+0.0215	0.9995	0.2032–203.2	0.2032
Midazolam	*Y* = 0.0725*X*+0.0366	0.9968	0.5000–500.0	0.5000

### 3.2 Effects of VDL on CYP450 activities in rats

**Fig 2:**
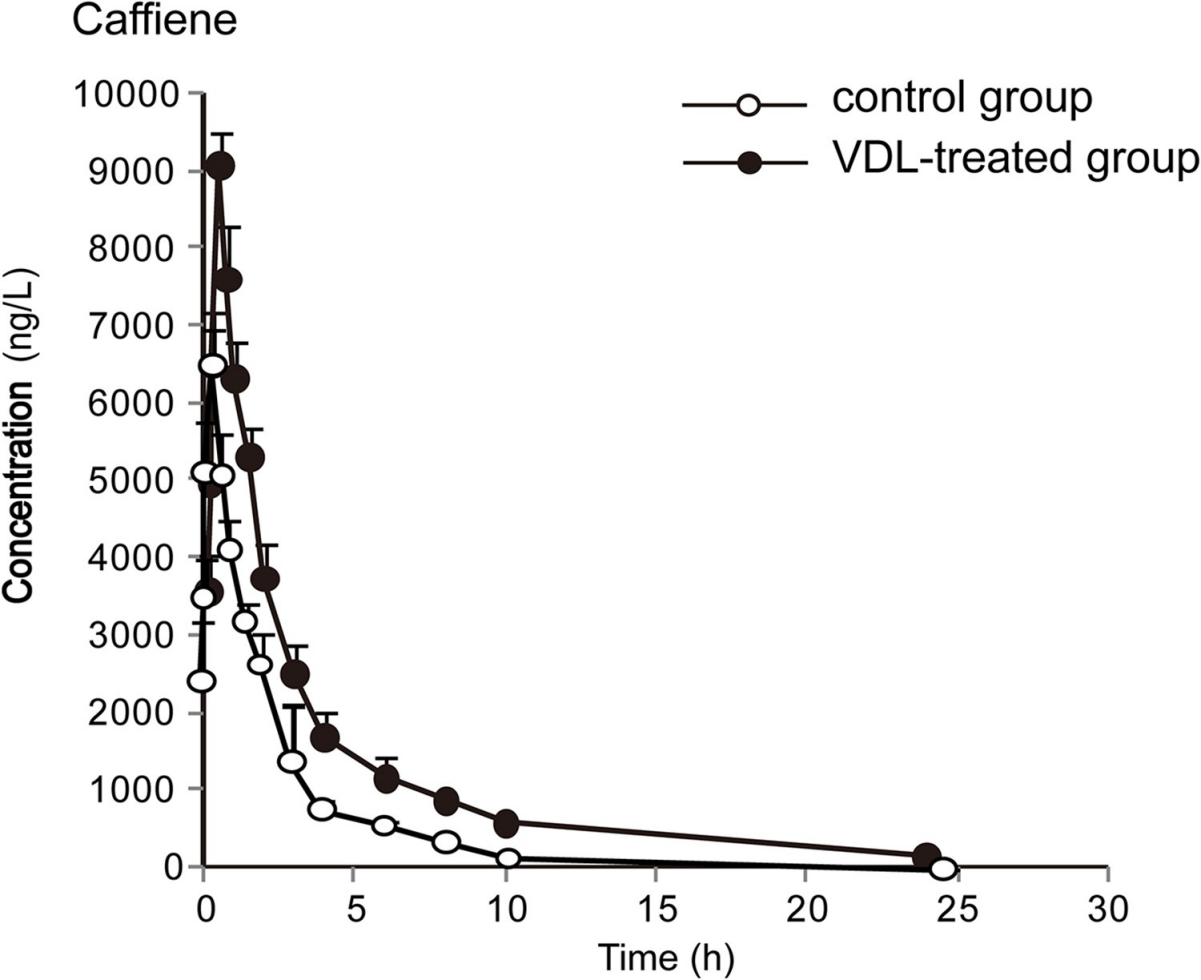
The plasma concentration-time curves of caffeine in rats ($\bar{x}$ ± *s*, n = 7).

**Fig 3:**
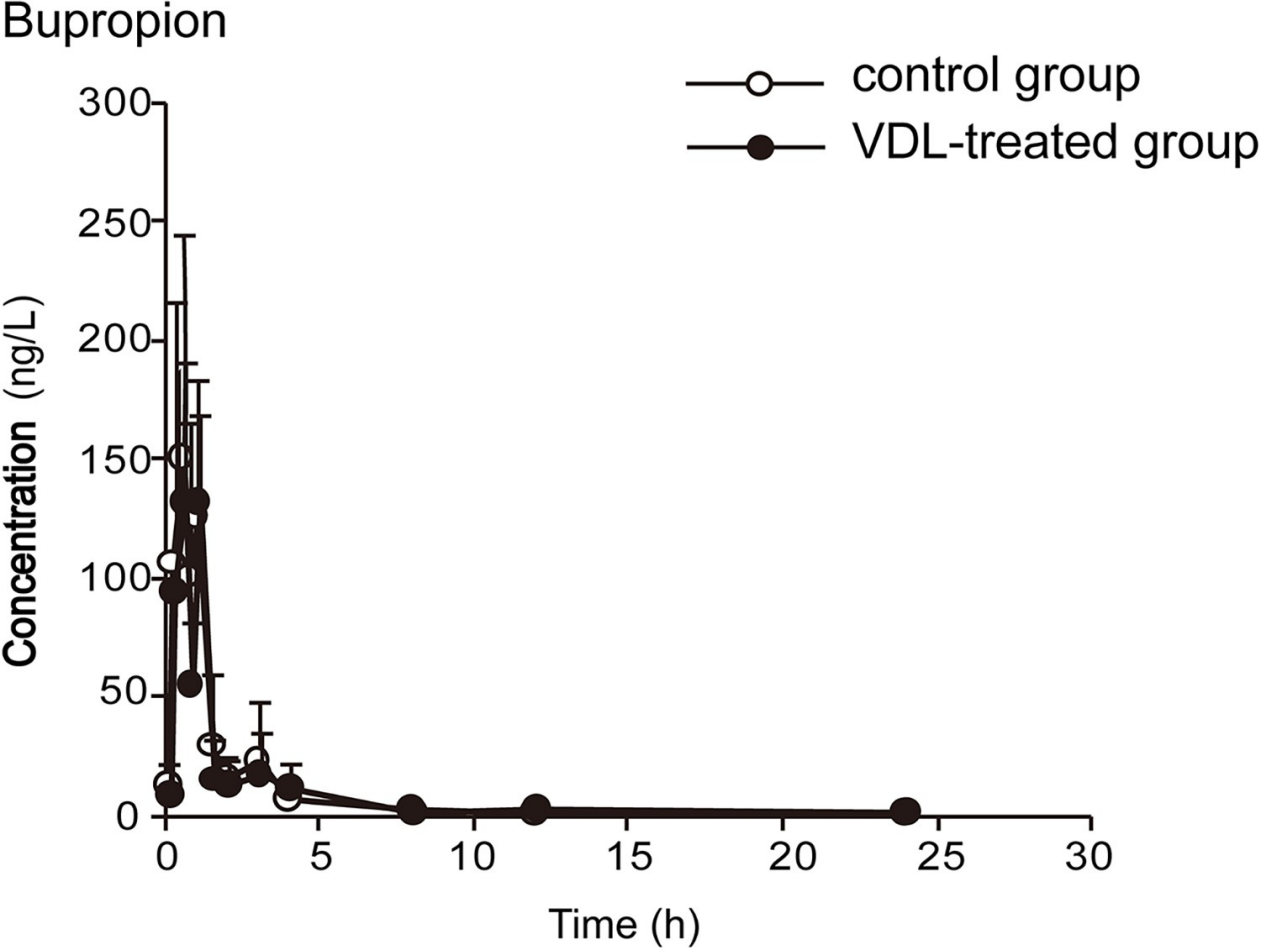
The plasma concentration-time curves of bupropion in rats ($\bar{x}$ ± *s*, n = 7).

**Fig 4:**
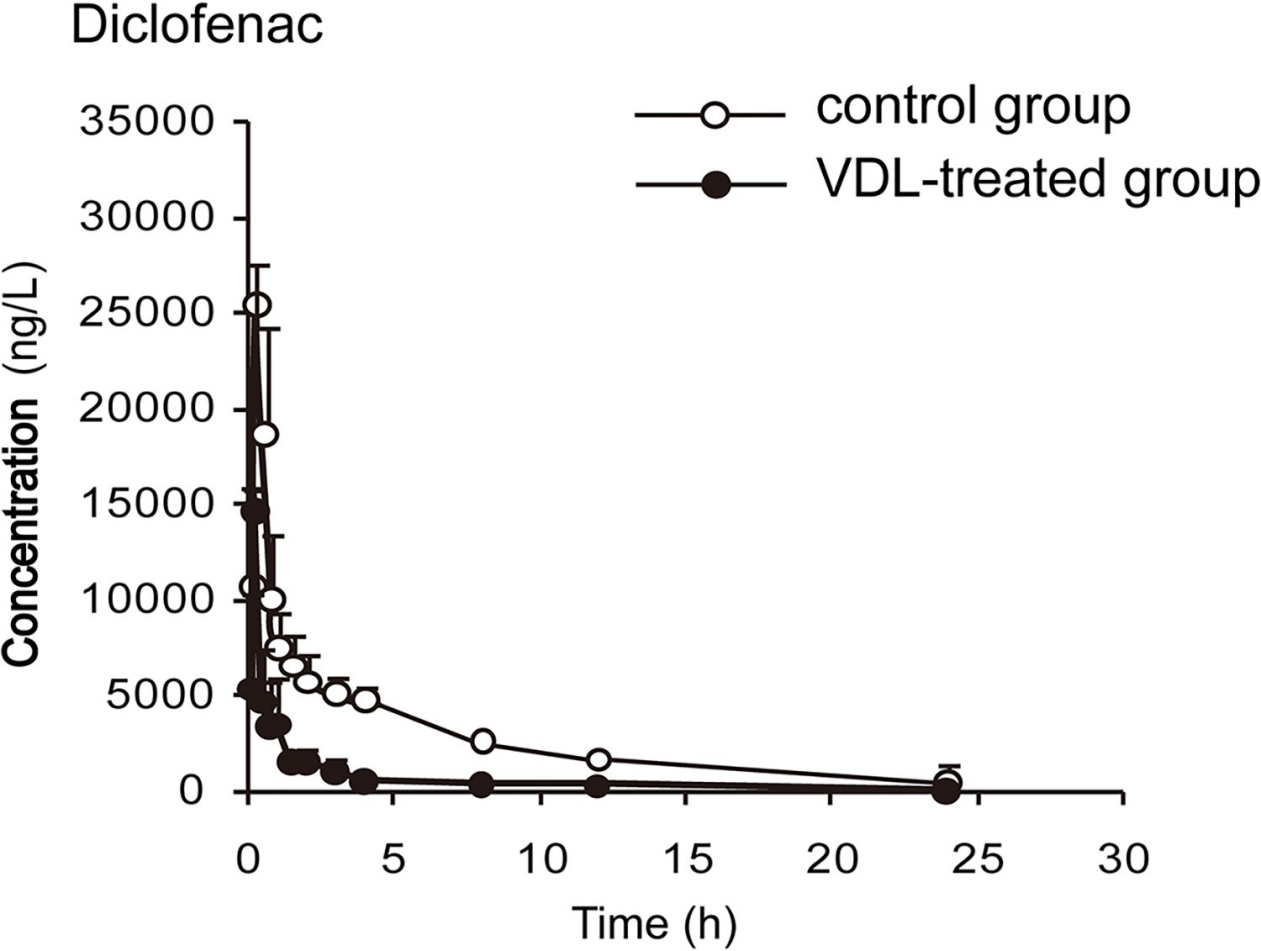
The plasma concentration-time curves of diclofenac in rats ($\bar{x}$ ± *s*, n = 7).

**Fig 5:**
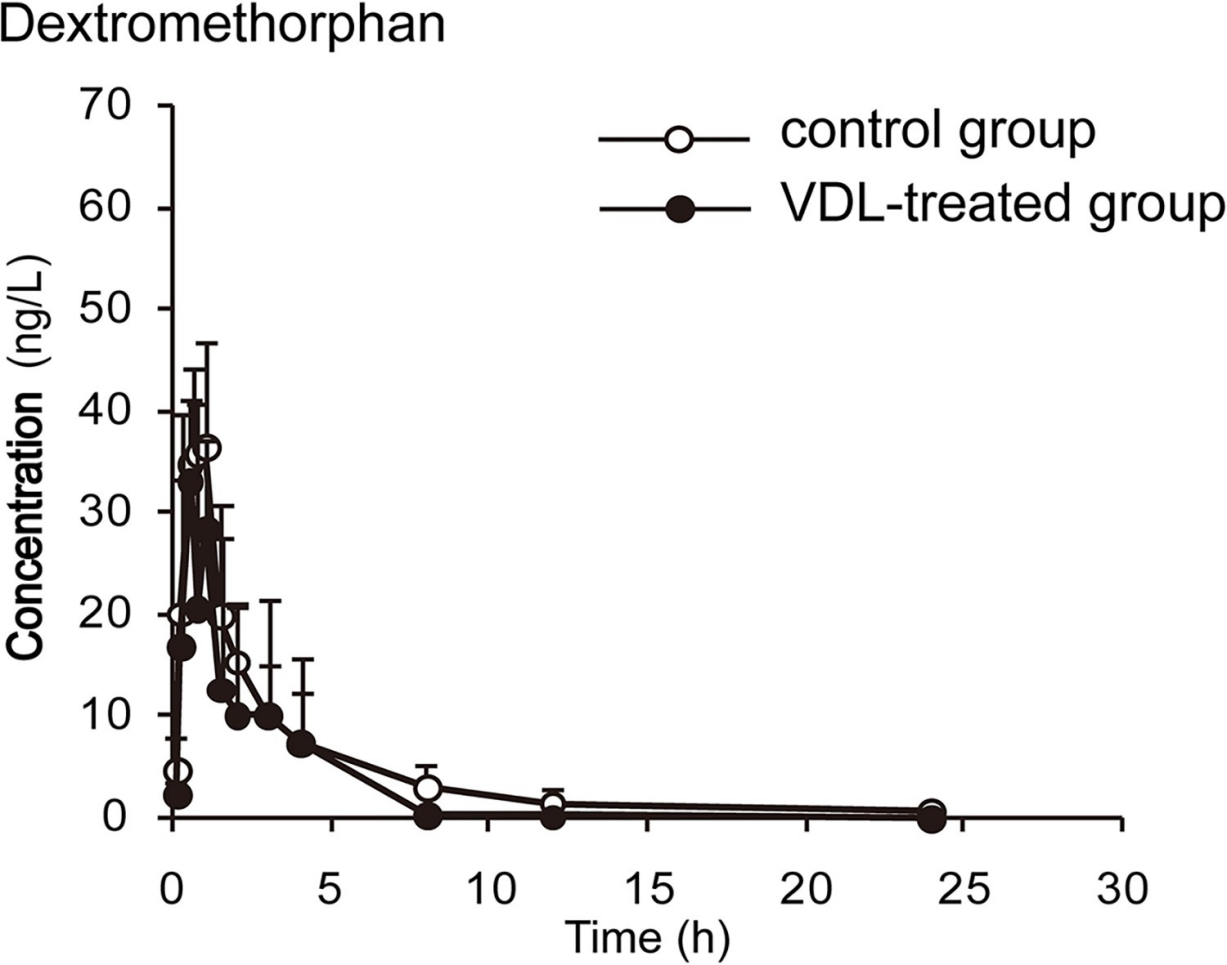
The plasma concentration-time curves of dextromethorphan in rats ($\bar{x}$ ± *s*, n = 7).

**Fig 6:**
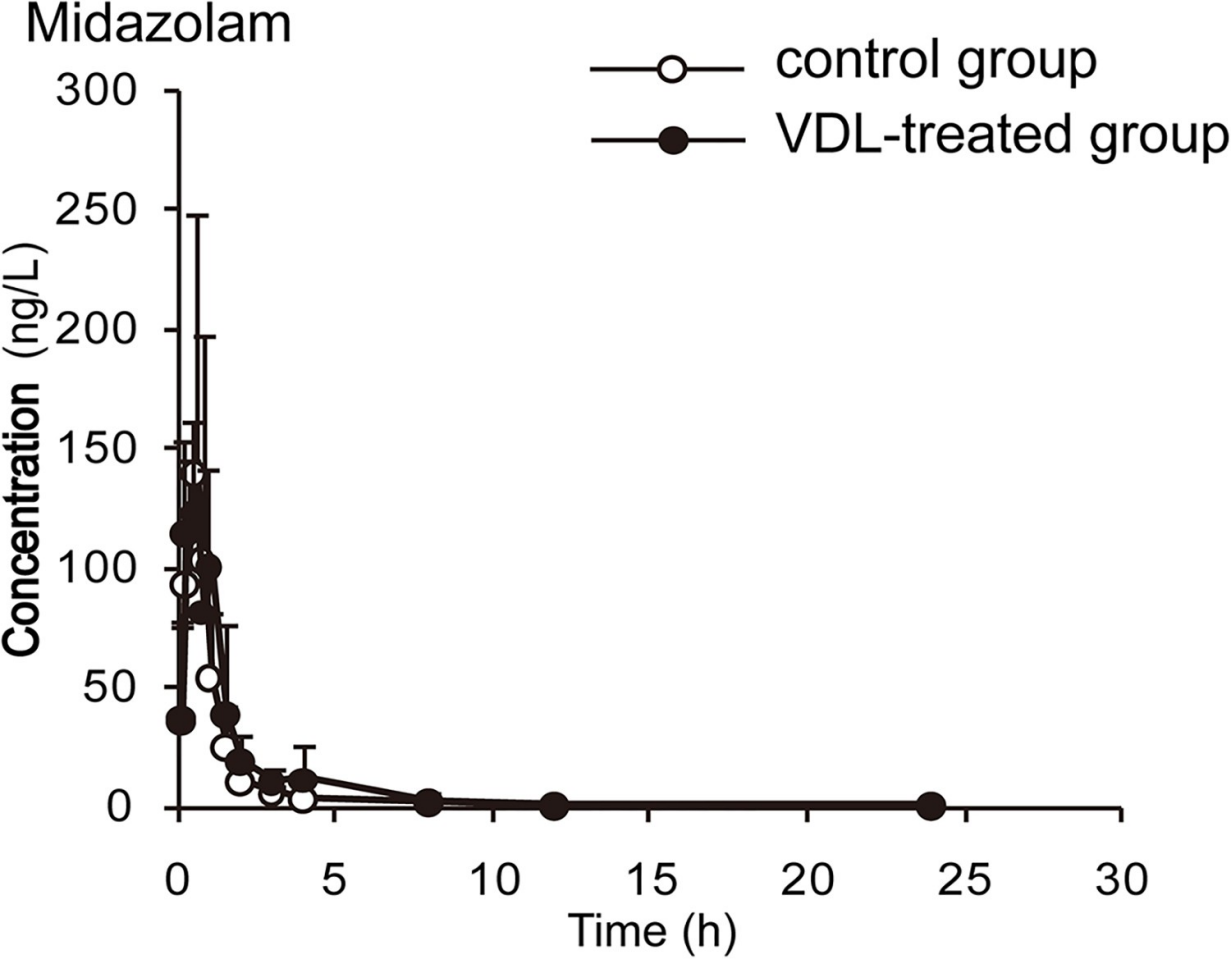
The plasma concentration-time curves of midazolam in rats ($\bar{x}$ ± *s*, n = 7).

The concentration-time curves and pharmacokinetic parameters are shown in Figs 2–6 and Tables 3–7, respectively.

**Table 3:** Main pharmacokinetic parameters of 1A2 probe after VDL treatment in rat plasma.

Parameter	Control group	VDL group
*t*_*1/2*_(h)	3.531±0.891	6.318±0.965*
*t*_*max*_(h)	0.550±0.224	0.631±0.198
*C*_*max*_(μg/L)	6616.750±460.231	9036.980±433.138*
*CL*(L/kg/h)	0.561±0.114	0.298±0.201*
*AUC*_*0-t*_(μg/L*h)	16631.066±2206.213	27694.576±1800.691*
*AUC*_*0-∞*_(μg/L*h)	17212.233±2388.725	28529.112±1680.953*

Data shown: Mean ± SD, n = 6

*: P<0.05, significantly different from CON.

**Table 4:** Main pharmacokinetic parameters of 2B probe after VDL treatment in rat plasma.

Parameter	Control group	VDL group
*t*_*1/2*_(h)	3.984±2.502	4.318±4.115
*t*_*max*_(h)	0.750±0.224	0.600±0.285
*C*_*max*_(μg/L)	196.197±139.950	130.139±109.543
*CL*(L/kg/h)	54.422±29.986	84.293±43.897
*AUC*_*0-t*_(μg/L*h)	224.914±127.400	139.095±94.039*
*AUC*_*0-∞*_(μg/L*h)	231.229±133.884	151.432±94.039

Data shown: Mean ± SD, n = 6

*: P<0.05, significantly different from CON.

**Table 5:** Main pharmacokinetic parameters of 2C11 probe after VDL treatment in rat plasma.

Parameter	Control group	VDL group
*t*_*1/2*_(h)	5.168±4.121	3.221±2.572*
*t*_*max*_(h)	0.421±0.122	0.376±0.675
*C*_*max*_(μg/L)	27019.055±4470.845	14063.362±15813.797*
*CL*(L/kg/h)	0.202±0.082	0.419±0.195*
*AUC*_*0-t*_(μg/L*h)	24102.91±9338.088	12364.838±5449.372*
*AUC*_*0-∞*_(μg/L*h)	26049.697±8586.410	13976.346±5213.664*

Data shown: Mean ± SD, n = 6

*: P<0.05, significantly different from CON.

**Table 6:** Main pharmacokinetic parameters of 2D1 probe after VDL treatment in rat plasma.

Parameter	Control group	VDL group
*t*_*1/2*_(h)	4.609±3.081	3.754±1.994
*t*_*max*_(h)	1.208±0.641	0.750±0.447
*C*_*max*_(μg/L)	45.521±27.184	36.548±41.193
*CL*(L/kg/h)	56.952±28.582	99.807±64.716*
*AUC*_*0-t*_(μg/L*h)	110.450±66.832	80.144±70.492
*AUC*_*0-∞*_(μg/L*h)	114.946±68.844	96.197±92.786

Data shown: Mean ± SD, n = 6

*: P<0.05, significantly different from CON.

**Table 7:** Main pharmacokinetic parameters of 3A probe after VDL treatment in rat plasma.

Parameter	Control group	VDL group
*t*_*1/2*_(h)	4.561±2.549	3.309±2.941
*t*_*max*_(h)	0.625±0.209	0.750±0.500
*C*_*max*_(μg/L)	147.785±105.240	105.354±100.109
*CL*(L/kg/h)	43.942±28.535	61.610±39.874
*AUC*_*0-t*_(μg/L*h)	156.490±87.649	124.865±94.581
*AUC*_*0-∞*_(μg/L*h)	158.789±88.139	138.451±114.184

Data shown: Mean ± SD, n = 6

*: P<0.05, significantly different from CON.

By comparing the pharmacokinetic parameters of blank group and VDL pretreatment group, VDL weakened the metabolism of caffeine, by increasing *t*_*1/2*_ (78.92%, *p*<0.05), *C*_*max*_ (36.78%, *p*<0.05), *AUC*_*0-t*_ (66.52%, *p*<0.05), *AUC*_*0-∞*_ (65.74%, *p*<0.05), and decreasing caffeine clearance (*CL*) (46.88%, *p*<0.05). In addition, VDL pretreatment accelerated the metabolism of diclofenac by decreasing *t*_*1/2*_ (37.67%, *p*<0.05), *C*_*max*_ (47.95%, *p*<0.05), *AUC*_*0-t*_ (48.70%, *p*<0.05), *AUC*_*0-∞*_ (51.79%, *p*<0.05) and increasing diclofenac clearance (*CL*) (51.79%, *p*<0.05). But no significant differences were observed in the major pharmacokinetics parameters of bupropion, dextromethorphan and midazolam between the VDL group and the blank group, which meant that there were no effects of VDL on CYP2B, CYP2D1 and CYP3A.

## 4. Discussion

### 4.1 Cocktail probe drugs method

The cocktail probe drugs method is a screening tool to predict the enzyme activity. In 1990, Breimer, DD *et al*. first used multiple probe substrates simultaneously to evaluate the activity of multiple CYP450 subtypes of enzymes, which called “Cocktail probe drug approach”. This approach is capable of measurement the activity of multiple CYP enzymes after the administration of multiple CYP-specific substrates in a single experiment.

The V_max_ and K_m_ values of cocktail probe drug approach and single probe approach are consistent, thus cocktail probe drug approach can replace single probe approach for the evaluation of CYP450 activity [25]. So far, the cocktail method can allow fast routine measurement of the multiple CYP enzyme activities in a single experiment, and this method also can minimize the confounding influence of inter-individual and intra-individual variability [21, 26, 27]. Therefore, the cocktail method is a powerful tool for comprehensively evaluating drug interactions involving multiple CYP enzymes simultaneously.

According to the “Drug Interaction Research” guidelines released by the FDA in the United States [28], the selection of *in vivo* study probe drugs depends on the CYP450 enzyme. Generally, probe drugs that specifically bind to isoenzymes should be selected. Therefore, according to the recommended guidelines, we have chosen the following probe drugs: (1) Midazolam (CYP3A); (2) Caffeine (CYP1A2); (3) Bupropion (CYP2B); (4) Diclofenac (CYP2C9); (5) Dextromethorphan (CYP2D6). In the experiment, the selection of probe drug dosage is also very important. The dose range of the probe drug was determined through literature [29–31], then preliminary experiments were conducted to determine the actual dosage, ensuring that low doses of composite probes were administered to experimental animals within the allowable range of detection capabilities, which can reduce the possibility of potential drug-drug interactions between probe drugs [21]. Finally, we determine the dose of the probe drugs, which is caffeine 5 mg·kg ^-1^, bupropion 5 mg·kg ^-1^, diclofenac 5mg·kg ^-1^, dextromethorphan 5mg·kg ^-1^, midazolam 5 mg·kg^-1^.

### 4.2 Induction and inhibition of CYP450 isoform activity by VDL

In the treatment of diabetes, patients usually need to take multiple drugs, and the combination of multiple drugs is very likely to cause DDIs [32]. If several drugs are metabolized through the same enzyme, the drug efficacy will increase or decrease, which will lead to treatment failure [33]. As a potential anti diabetes drug, it is necessary to study the effect of VDL on enzyme activity. In this study, we found that VDL tended to be the inducer of CYP2C11 (CYP2C9 in human). Stephen S. *et al*. reported that the nuclear receptor CAR (Constitutive androgen receptor) as an effective regulator of CYP2C9 transcription at both the mRNA level and in promoter assays [34]. So we think that VDL might upregulate the transcription level of CYP2C9 through the CAR receptor. In addition, we found that VDL had an inhibitive effect on CYP1A2. It is reported that nitrogen-containing heterocyclic compounds can bind tightly to the prosthetic haem iron of CYP proteins; this binding often exhibits reversible inhibition on CYP enzymes [35]. VDL is a single indole alkaloid, which have nitrogen-containing pyrrole rings. So we think that the inhibitory effect of VDL on CYP1A2 may be related to the nitrogen-containing heterocycles in VDL.

The induction of CYP leads to the increase of the synthesis or activity of CYP, thus accelerating the metabolism of drugs eliminated by this procedure, leading to the reduction of plasma drug concentration and treatment delay [36]. In this study, treatment of rats with VDL decreased the *t*_*1/2*_, *C*_*max*_, *AUC*_*0-t*_, *AUC*_*0-∞*_ and increased clearance (*CL*) of diclofenac compared with those in the blank control. It is suggested that VDL could induce CYP2C11, which is homolog to human CYP2C9. CYP2C9 is one of the most important metabolic enzymes in the CYP enzyme family. About 15% of clinical drugs are metabolized by CYP2C9, including celecoxib, phenytoin sodium, diazepam, tolbutamide, diclofenac acid, losartan and S-warfarin [37, 38]. In clinic, when applied in combination, VDL may interact with all drugs metabolized by CYP2C9, especially for drugs with a narrow therapeutic window, such as phenytoin sodium. Therefore, VDL should be avoided in combination with these drugs.

According to the experimental results, the activity of CYP1A2 might be inhibited by VDL. Enzyme inhibition refers to those drugs can inhibit the activity of enzymes during the combined use of drugs, resulting in slower metabolism of the combined drugs, making the concentration of drugs in the body higher than the normal concentration when used alone, and enhancing the effect [35]. For drugs with narrow treatment window, serious adverse reactions may occur [39]. CYP1A2 accounts for only 13% in the CYP enzyme family, but many important marketed drugs are metabolized by CYP1A2 [40]. In the future clinical application of VDL, it is likely to be combined with many drugs, including antihypertensive drugs, hypoglycemic drugs, and lipid-lowering drugs and so on [41], if the main metabolic enzyme of these drugs is CYP1A2, because VDL has an inhibitory effect on CYP1A2, the plasma concentration of these drugs will increase compared with the concentration when they are used alone, which can cause adverse effects, and may endanger life in serious cases [42, 43].

### 4.3 Analysis of in vitro and in vivo experimental results

In the previous *in vitro* experiments, it was found that VDL could inhibit CYP2D1 activity, and had weak inhibitory effect on CYP2C11 and CYP3A (S1 Fig), which was inconsistent with the results of *in vivo* experiments [44]. The reasons for this phenomenon may be as follows: (1) Influence of *in vivo* and *in vitro* action time and dosage: *in vitro* experiments, VDL and probe drugs were directly incubated with CYP450 enzymes in rat liver microsome, while *in vivo* experiments, rats were pretreated with VDL for 15 days, and then the probe substrate was administered on the 16 days. The expression environment of the enzyme, and the dosage in rat are differences, it may cause VDL to induce or inhibit the CYP450 enzyme in different ways. (2) Compared with *in vivo* experiment, the environment of the enzymatic reaction was artificially simulated *in vitro*, and the interference of other complex factors in the body was excluded. But *in vivo* experiments reflect the metabolism of drugs in the physiological state of the whole living organism. This difference of *in vitro* and *in vivo* environment may cause changes in the activity and quantity of CYP450 enzymes, which may lead to inconsistencies in the results of *in vitro* and *in vivo* experiments [45]. This study suggested that examining the effects of drugs on CYP450 enzyme activity, *in vitro* and *in vivo* experiments are equally important. Preliminary exploration of *in vitro* experiments can provide ideas for further *in vivo* experiments, but whether the *in vitro* experimental data can be used to directly predict the interaction results *in vivo* remains to be further verified.

## 5. Conclusion

In this study, the *in vivo* results was showed that VDL inhibited the activity of CYP1A2, and induced the activity of CYP2C9, but had no effects on the activities of CYP2B, CYP2D6 and CYP3A4. Based on the above findings, we need follow with interest potential DDIs regulated by CYP1A2 and 2C9, when VDL combined with other drugs (particularly for drugs with narrow therapeutic window). However, the relevant research and clinical significance of human still need further study.

## Supporting information

S1 FigThe inhibition curves of VDL on five CYP450 isoforms ($\bar{x}$ ± *s*, n = 6).(TIF)
