# Supplementary figures and images for: Evaluation of the impact of vindoline, an active components of Catharanthus roseus, on rat hepatic cytochrome P450 enzymes by using a cocktail of probe drugs
Source: PLoS One. 2023 Aug 3;18(8):e0289656. doi: 10.1371/journal.pone.0289656 (PMC10399899; doi:10.1371/journal.pone.0289656)

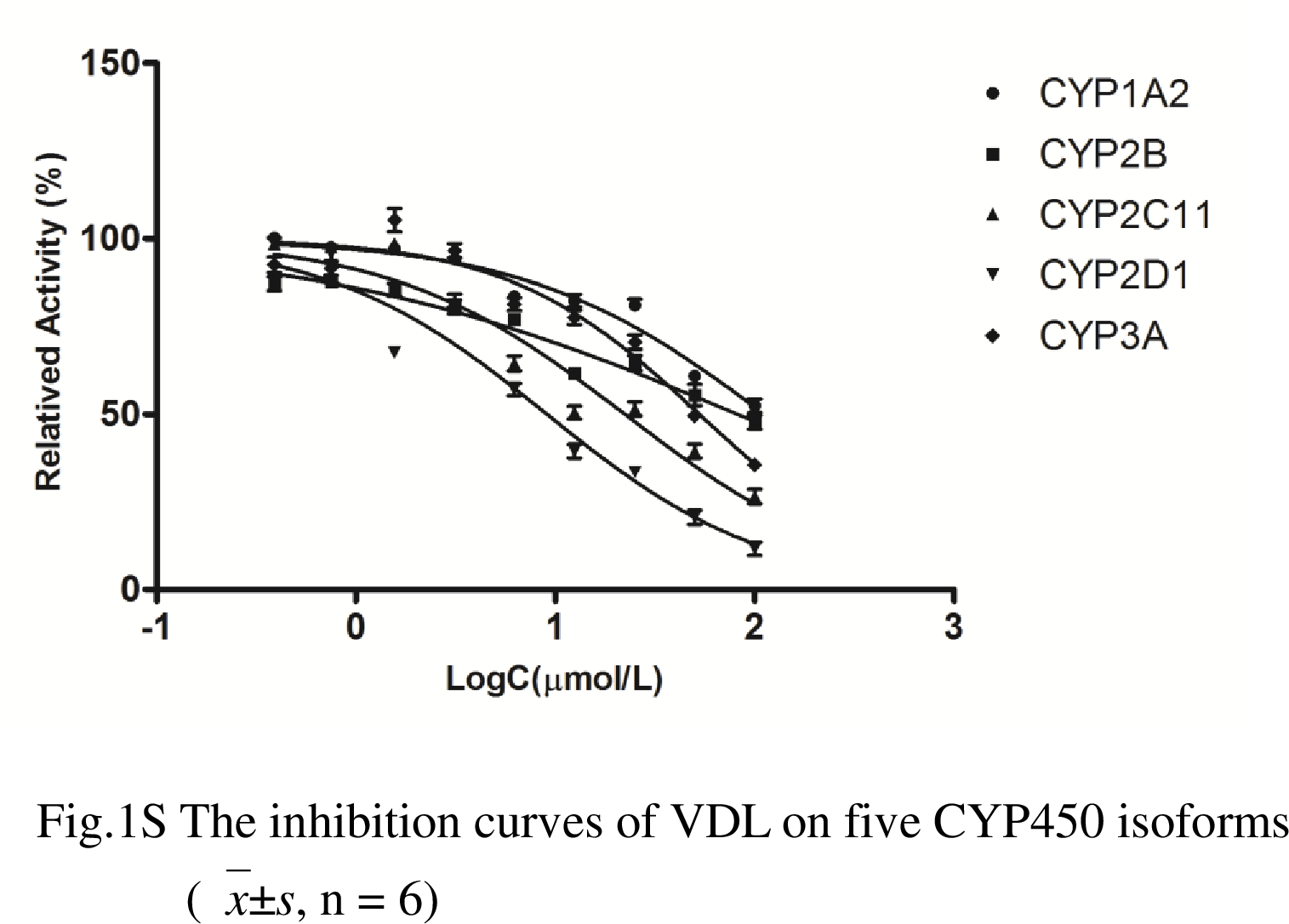

Supplement: S1 Fig — (TIF) [file pone.0289656.s001.tif]
